# Noise and Complexity in Human Postural Control: Interpreting the Different Estimations of Entropy

**DOI:** 10.1371/journal.pone.0017696

**Published:** 2011-03-17

**Authors:** Christopher K. Rhea, Tobin A. Silver, S. Lee Hong, Joong Hyun Ryu, Breanna E. Studenka, Charmayne M. L. Hughes, Jeffrey M. Haddad

**Affiliations:** 1 Department of Kinesiology, University of North Carolina at Greensboro, Greensboro, North Carolina, United States of America; 2 Department of Kinesiology, University of Massachusetts, Amherst, Massachusetts, United States of America; 3 Department of Kinesiology, Indiana University, Bloomington, Indiana, United States of America; 4 Department of Health and Kinesiology, Purdue University, West Lafayette, Indiana, United States of America; 5 Department of Kinesiology, The Pennsylvania State University, University Park, Pennsylvania, United States of America; 6 Neurocognition and Action Research Group, Faculty of Psychology and Sport Sciences, Bielefeld University, Bielefeld, Germany; 7 Center for Aging and the Life Course, Purdue University, West Lafayette, Indiana, United States of America; University of Maribor, Slovenia

## Abstract

**Background:**

Over the last two decades, various measures of entropy have been used to examine the complexity of human postural control. In general, entropy measures provide information regarding the health, stability and adaptability of the postural system that is not captured when using more traditional analytical techniques. The purpose of this study was to examine how noise, sampling frequency and time series length influence various measures of entropy when applied to human center of pressure (CoP) data, as well as in synthetic signals with known properties. Such a comparison is necessary to interpret data between and within studies that use different entropy measures, equipment, sampling frequencies or data collection durations.

**Methods and Findings:**

The complexity of synthetic signals with known properties and standing CoP data was calculated using Approximate Entropy (ApEn), Sample Entropy (SampEn) and Recurrence Quantification Analysis Entropy (RQAEn). All signals were examined at varying sampling frequencies and with varying amounts of added noise. Additionally, an increment time series of the original CoP data was examined to remove long-range correlations. Of the three measures examined, ApEn was the least robust to sampling frequency and noise manipulations. Additionally, increased noise led to an increase in SampEn, but a decrease in RQAEn. Thus, noise can yield inconsistent results between the various entropy measures. Finally, the differences between the entropy measures were minimized in the increment CoP data, suggesting that long-range correlations should be removed from CoP data prior to calculating entropy.

**Conclusions:**

The various algorithms typically used to quantify the complexity (entropy) of CoP may yield very different results, particularly when sampling frequency and noise are different. The results of this study are discussed within the context of the neural noise and loss of complexity hypotheses.

## Introduction

Complex dynamic patterns, present in virtually all physiological processes, arise from the interaction between the organism and its environment, both of which fluctuate constantly [1]–[2]. Examining the degree of chaos or complexity in a system has proven to be a valuable and increasingly useful tool in the study of human health [3]–[6]. The loss of complexity in aging was first demonstrated by lower values of Approximate Entropy (ApEn) [3] in the dynamics of heartbeat intervals in frail elderly compared to young participants [4]. Since then, numerous studies using a variety of different entropy measures have demonstrated that complexity is lost in various biophysical signals (e.g., hormonal patterns, blood pressure, human postural control) as a result of aging, disease and/or disorder. Complex and chaotic patterns are different from random noise because they can be modeled using completely deterministic equations. However, it is often difficult to determine if fluctuations in human physiological data are chaotic or the result of random neuromuscular noise [1]. Effectively, determining “what is random?” in biological data remains a widely debated topic [7], [8].

Human postural control provides a unique opportunity to test how determinism and noise influence a physiological signal. This is because the control of upright posture requires the integration of a variety of sensory signals and the coordinated contraction of numerous muscles. Postural control is typically studied by examining the dynamic patterns in center of pressure (CoP) trajectories while standing on a force platform. *Internal* neuromuscular noise and *external* noise from the force platform will both be inherent in the signal. Physiologically, it has been suggested that neural noise is the mechanism that leads to variability in behavioral performance [9]–[11] and that age-related increases in neural noise leads to deficits in performance [12]. Mechanically, the degree of external noise can vary depending on the type of force platform used (e.g., strain gauge vs. piezoelectric vs. Hall effect) and the data collection environment. Regardless of whether noise originates from physiological or mechanical factors, it is unknown how different levels of noise affect various measures of entropy. Thus, it is often difficult to interpret complexity changes in a biological system.

Direct measures of entropy, such as Kolmogorov-Sinai entropy, can only be used on time series data that contain a very large number of noise free data points [3]. CoP data is never noise free and typically relatively short to minimize the effects of fatigue and inattention. Therefore, direct measures of entropy cannot be used. As a result, a variety of indirect measures that estimate entropy and are more robust to time series length and noise have been developed. For example, ApEn has been showed to accurately discriminate between clinically distinct cohorts with short time series with as few data points as N = 144 [13]. These indirect measures are all termed ‘entropy,’ but each uses a different algorithm to estimate the complexity of a time series. ApEn [3] and Sample Entropy (SampEn) [5] are approximations of Kolmogorov-Sinai entropy and calculate the likelihood that a template pattern repeats in the time series. Alternatively, Recurrence Quantification Analysis Entropy (RQAEn) is derived from Shannon entropy and is calculated by examining the number of line segments of varying length in a recurrence plot [6]. Although these measures are named “entropy”, fundamental differences in their algorithms make their similarities mostly semantic. Thus, comparisons and interpretations across studies are difficult. Understanding the properties of each analysis method is critical in deciding whether the interpretation of pathological movements observed in a given disorder should be inferred based on the neural noise or loss of complexity hypothesis.

Another potential confound in the estimation of entropy in a postural sway signal is the frequency at which the data are sampled. It has been suggested that the fastest voluntary movement a human can produce is 8–10 Hz [14]. Based on the Nyquist theorem, a sampling frequency greater than 20 Hz would be sufficient to provide an alias-free signal of postural sway and there would be no further advantage of increasing the sampling frequency when examining the dynamics of the behavior. However, postural control occurs at a variety of time scales [15], so lower sampling frequencies may not provide an accurate record of the system's dynamics. Conversely, oversampling could lead to co-linearities in the signal [16], thus artificially affecting the dynamics.

Additionally, the length of a time series (number of data points used in the calculation) has been shown to influence measures of entropy [5]. The length of a time series can obviously vary between studies if different sampling frequencies are used. In this case, entropy measures may vary because more data points were used in the entropy calculation or because (as discussed above) shorter time scale dynamics were captured. Time series length can also vary when data is collected over different intervals of time. Collecting for a longer interval of time (at a set sampling frequency) will increase the number of data points used in the entropy calculation and also increase the likelihood that transient movement dynamics, not related to a steady state, are captured. For example, when collecting CoP data, if a participant shifts their body weight during a trial, it will change the dynamics of the signal. Since these types of movements are often the result of fatigue or a loss of attention, they are most likely to occur at the end of a trial. Therefore, studies that collect for a long duration of time may capture different dynamics compared to studies utilizing trials that occur over shorter time intervals. Hence, different sampling frequencies or different time series lengths, a seemingly small difference from one study to the next, could have a significant influence on the calculation of entropy and subsequent interpretation of the data. Thus, when comparing data between studies, both data collection time and sampling frequency should be carefully considered. Within a study, these issues are not as problematic since sampling frequency and data collection time are typically constant. One exception is in studies where CoP is captured during a transient movement (e.g. while reaching for an object). In these types of studies, data is often analyzed from the start to the end of the reach. Since reach duration can change from one trial and condition to the next, data collection time can also vary.

The influence of noise and sampling frequency on three estimates of entropy (ApEn, SampEn and RQAEn) was examined by comparing synthetic signals against CoP data. Specifically, the influence of noise was examined using three deterministic signals with known noise properties and by adding white noise to CoP signals. The influence of sampling frequency was examined by downsampling the original CoP signal that was collected at 1000 Hz. The sampling frequency manipulation was also used to yield insight regarding how time series length influences the various entropy calculations. We hypothesized that an increase in noise and sampling frequency would independently lead to higher entropy values (i.e., increased complexity).

## Methods

### Participants and experimental procedure

Six participants (three males and three females; *M* age: 25.6±4.8 yrs; *M* mass 73.7±18.0 kg) gave written informed consent prior to data collection. The methodology and consent form for this study were approved by the Purdue University Institutional Review Board. All participants were free of any neurological conditions that would influence balance. Each participant stood on a force platform with eyes open, feet shoulder width apart and arms comfortably at their side for 30 seconds. CoP data were collected at 1000 Hz using one AMTI force platform (Watertown, MA, USA).

### General data processing

In typical quiet standing, the majority of CoP displacement occurs in the anterior-posterior (AP) direction. Thus, only data in the AP direction were analyzed. To ensure the analyzed data were recorded when the participants were in a steady postural state, only the middle eight seconds (8000 data points) of the time series was used in the subsequent analysis. It has recently been suggested that examining the dynamics of CoP is inappropriate due to the presence of long-range correlations [17], which have been shown to potentially mask the complexity of a time series [18]. To correct for this issue, an increment (or difference) time series created from the original signal was used to remove long-range correlations [18]. The increment time series was created by calculating the difference between each data point (i.e., [*x*(*t*+1) – *x*(*t*)]) in the original time series [17]. Roerdink et al. [19] found differences in SampEn between the original and increment CoP data in sitting and standing posture. However, it is unknown how ApEn and RQAEn change due to the increment transformation. Therefore, the original and increment CoP data were examined in all entropy analyses (further described below).

### Influence of noise on synthetic signals

**Figure 1:**
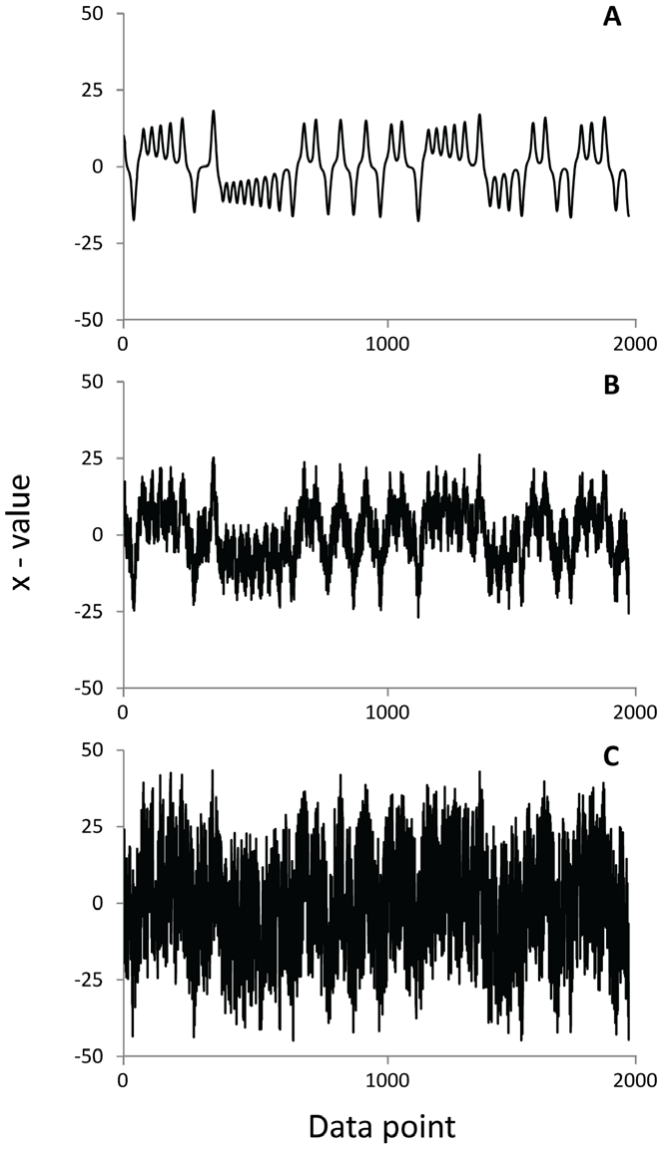
The Lorenz attractor with additive noise. The first 2000 points of the x-value of the Lorenz attractor using parameters of σ = 10, β = 8/3 and ρ = 28 for the deterministic (A), semi-deterministic (B) and stochastic (C) synthetic signals.


The influence of noise on entropy was examined using synthetic signals with known properties that are theoretically related to postural control. There is disagreement in the literature as to the type of noise found in a CoP signal. Some have suggested that postural control is a chaotic process [20] while others have suggested that the process is stochastic [21], [22]. To account for these possibilities, three signals (chaotic [completely deterministic], semi-deterministic and stochastic [non-deterministic]) were created (Figure 1). The alpha value in detrended fluctuation analysis (DFA) [23] was used to quantify the long-range correlations in each synthetic signal, which relates to amount of noise. The first signal (chaotic; DFA α = 1.28) consisted of the first 2000 points in the time series from the x-value of the Lorenz attractor with a one point time step, which is specified by the three following equations:

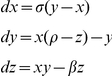


Parameters were set to σ = 10, ρ = 28 and β = 8/3. The second signal (semi-deterministic) was created by adding white noise to the chaotic signal using the Jitter function in Matlab, which multiplies all data points by the smallest distance between any two points in the time series (scaling factor µ). Using a µ = 5000 units, the chaotic signal was transformed into a semi-deterministic signal (DFA α = 1.02). Lastly, µ = 15,000 units of white noise were added to the chaotic signal to create a stochastic signal (DFA α = 0.73).

### Influence of noise and sampling frequency on CoP signals

The influence of noise and sampling frequency on CoP entropy was examined by adding µ = 40–200 units of white noise (in 40 unit increments) to AP CoP signals at each of the sampling frequencies. The 1000 Hz time series was downsampled to create five new time series with the effective sampling rates of 500, 333, 250, 200 and 166 Hz in both the original and increment data, yielding 25 new signals (5 noise levels by 5 sampling frequencies). By altering sampling frequency and maintaining a constant recording duration, the length of the time series was also manipulated. A two-way repeated measures ANOVA was used for each entropy estimate to determine whether entropy changed with increased noise at the different sample frequencies. Follow-up protected *t*-tests were used when appropriate. Significance was set at *p*≤0.05 for the ANOVA, while a more conservative value of *p*≤0.01 was used for the follow-up tests to control for multiple pair-wise comparisons.

### Influence of sampling frequency independent of noise

To determine the influence of sampling frequency independent of artificial noise, the original and increment CoP data with no added noise (µ = 0) were independently examined at each of the sampling frequencies. A repeated measures within-subjects ANOVA was used to determine differences between each entropy estimate as a function of sampling frequency. Follow-up protected *t*-tests were used when appropriate. Significance was again set at *p*≤0.05 for the ANOVA, with a more conservative value of *p*≤0.01 used in the follow-up tests.

### Entropy calculations

ApEn, SampEn and RQAEn were calculated in Matlab for each of the signals using the algorithms published by Pincus [3], Richman and Moorman [5] and Weber and Zbilut [6], respectively. Computing ApEn, SampEn and RQAEn requires the selection of *m*, *r* and τ as input parameters. These are commonly referred to as “embedding dimension”, “radius” and “time delay”, respectively. Although the input parameters share the same terminology, their meaning within each entropy estimation algorithm is different. ApEn and SampEn measure the likelihood that a template pattern whose length (*m*) and criterion of similarity (*r*) at time delay (τ) will repeat in the time series. Conversely, RQAEn attempts to unfold the attractor landscape by looking for data points that recur within a radius (*r*) in multiple dimensions (*m*) separated by a time delay (τ). Although the entropy estimates require the “same” input parameters, their meaning is quite different since attractor reconstruction is part of RQAEn and not part of ApEn and SampEn.

For ApEn and SampEn, the template pattern's length and criterion of similarity were defined as *m* = 3 and *r* = 0.2 of the time series standard deviation, respectively. These values are consistent with previous studies that have examined the ApEn or SampEn of center of pressure [17], [24]–[27]. Measures of complexity exhibiting long-range correlations require the use of a time delay (τ) to accurately identify the dynamics of the system [18]. The strength of the long-range correlations varied between the synthetic signals, therefore entropy estimations were explored with a τ of 15 (appropriate for correlated signals) and 1 (appropriate for uncorrelated signals). A τ of 15 was used for the original CoP data. Long-range correlations should be removed by calculating the difference from point-to-point (which can be confirmed by DFA); therefore a τ of 1 was used with the increment CoP data.

To calculate RQAEn, the original CoP time series was reconstructed into a multidimensional state space using a τ of 15 data points and *m* of 3 as defined by the average displacement [28] and false nearest neighbors [29] techniques, respectively. A data point was counted as recurrent if it was within an *r* of 20% of the mean distance separating points in the reconstructed state space. Diagonal line structures in the recurrence plots were identified when two or more consecutive points were recurrent. RQAEn increases as more diagonal line structures of varying length are observed within the recurrence plot. RQAEn of the increment data was examined using the same parameters, with the exception of the τ parameter, which was set to 1.

Although different input parameters could have been used in each of the time series (especially the extremely noisy signals), it would have been impossible to determine if a change in the estimate of entropy was due to the signal's dynamics or to the change in the input parameters used in the calculation. Therefore, a consistent set of entropy parameters was used for the original and increment data sets in order to eliminate this issue.

## Results

**Figure 2:**
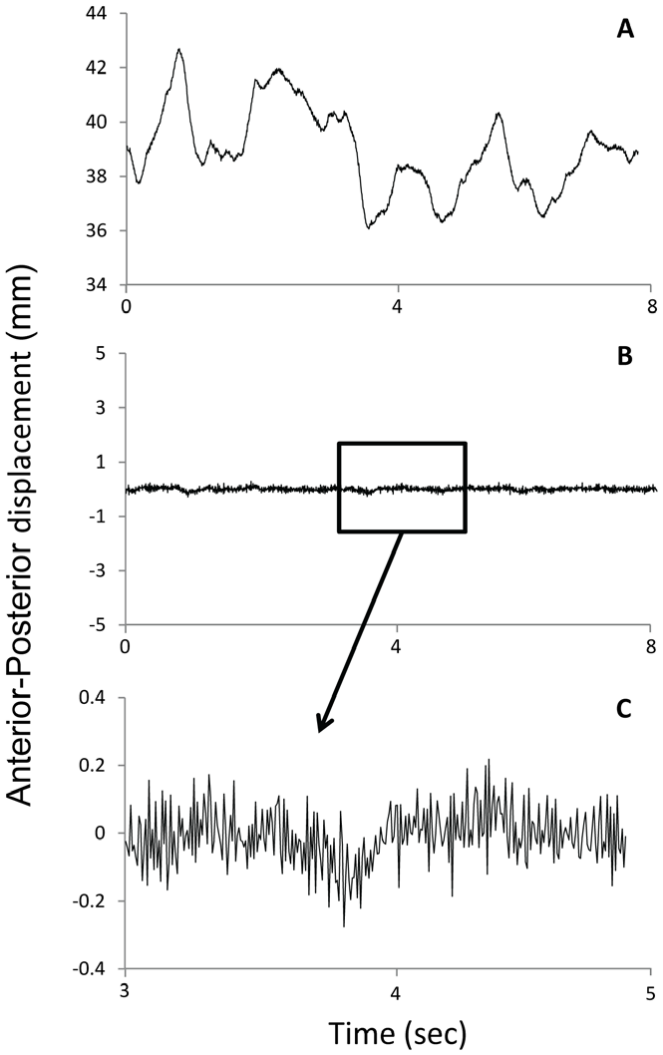
Example CoP plots. Example plot of the original center of pressure (CoP) time series from one subject with a sampling rate of 166 Hz (A), the corresponding increment CoP time series (B) and a zoomed in view of the increment CoP time series (C).

Long-range correlations were present in the original CoP data at all sampling frequencies and were successfully removed by the increment method (Table 1). The results for both the original and increment CoP time series (Figure 2) as a function of noise and sampling frequency are presented below.

**Table 1:** Detrended fluctuation analysis (DFA) alpha value for the original and increment center of pressure (CoP) data as a function of sampling frequency.

Sampling frequency	Original CoP	Increment CoP
500 Hz	1.65±0.08	0.58±0.17
333 Hz	1.63±0.06	0.64±0.15
250 Hz	1.60±0.08	0.65±0.14
200 Hz	1.57±0.09	0.66±0.13
166 Hz	1.54±0.11	0.65±0.12

### Influence of noise when calculating entropy using the synthetic signals

ApEn, with a time delay of both 1 and 15, exhibited an inverted-U function with increased noise. SampEn increased as noise increased with both time delays. The opposite was observed for RQAEn, where, entropy decreased with increasing noise (Table 2).

**Table 2:** Detrended fluctuation analysis alpha (DFA α), Approximate Entropy (ApEn), Sample Entropy (SampEn) and Recurrence Quantification Analysis Entropy (RQAEn) values for each synthetic signal with a time delay (τ) of 15 and 1.

	Chaotic signal	Semi-deterministic signal	Stochastic signal
DFA α	1.28	1.02	0.73
ApEn (τ = 15)	0.58	0.97	0.89
ApEn (τ = 1)	0.22	1.13	0.91
SampEn (τ = 15)	0.80	2.06	2.21
SampEn (τ = 1)	0.24	1.86	2.19
RQAEn (τ = 15)	3.93	0.11	0.00
RQAEn (τ = 1)	3.80	1.11	0.89

### Influence of noise and sampling frequency when calculating entropy using the CoP signals

**Figure 3:**
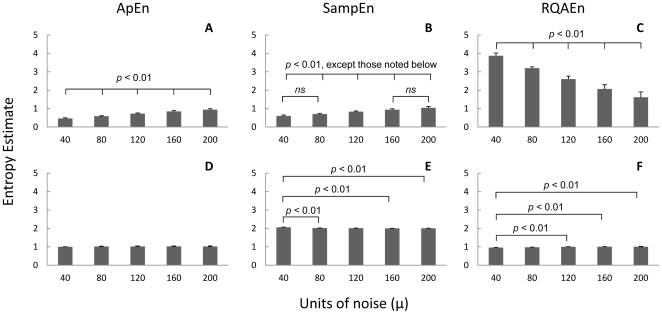
Influence of noise on CoP. Approximate Entropy (ApEn), Sample Entropy (SampEn) and Recurrence Quantification Analysis Entropy (RQAEn) as a function of added Gaussian noise for the original center of pressure (CoP) data (A, B and C) and the corresponding increment CoP data (D, E and F).

A noise by sampling frequency interaction was not observed in any of the entropy measures assessed using the original CoP data (all *p*>0.01). A main effect of noise was however observed for ApEn (*F*_4,20_ = 29.75, *p*<0.01), SampEn (*F*_4,20_ = 23.69, *p*<0.01) and RQAEn (*F*_4,20_ = 29.79, *p*<0.01) (Figure 3). A main effect of sampling frequency was also observed for ApEn (*F*_4,20_ = 8.08, *p* = 0.03), SampEn (*F*_4,20_ = 158.11, *p*<0.01) and RQAEn (*F*_4,20_ = 11.25, *p*<0.01). Follow-up tests for the noise main effect revealed the following: 1) ApEn systemically increased as noise increased (*p*≤0.01); 2) SampEn did not differ between signals with 40 and 80 units of noise (*p* = 0.012) or between signals with 160 and 200 units of noise (*p* = 0.013), but increased systemically as noise increased in all other signals (*p*≤0.01); and 3) RQA systematically decreased as noise increased (*p*≤0.01). Follow-up tests of the sampling frequency main effect revealed the following: 1) lower ApEn was observed in the 500 Hz signal compared to the 333 Hz and 250 Hz signals (both *p*<0.01), no other changes in ApEn were observed (*p*>0.01); 2) SampEn did not differ between the 250 Hz and 200 Hz signals (*p* = 0.02) or between the 200 Hz and 166 Hz signals (*p* = 0.03), but increased as sampling frequency decreased in all other signals (*p*<0.01); and 3) higher RQAEn was observed in the 500 Hz signal compared to the 250 Hz and 200 Hz signals (both *p*<0.01); no other changes in RQAEn were observed (*p*>0.01).

A noise by sampling frequency interaction was not observed when assessing ApEn, SampEn or RQAEn using the increment CoP data (all *p*>0.01). However, a main effect of noise was observed in ApEn (*F*_4,20_ = 8.81, *p* = 0.01), SampEn (*F*_4,20_ = 13.09, *p*<0.01) and RQAEn (*F*_4,20_ = 15.38, *p*<0.01) (Figure 3). A main effect of sampling frequency was also observed in ApEn (*F*_4,20_ = 2740.75, *p*<0.01), SampEn (*F*_4,20_ = 5.54, *p* = 0.03) and RQAEn (*F*_4,20_ = 11.18, *p*<0.01). Follow-up tests of the noise main effect revealed the following: 1) ApEn did not change as a function of noise level (*p*>0.01); 2) higher SampEn was observed in the signal with 40 units of noise compared to the signals with 80, 160 and 200 units of noise (*p*<0.01), no other changes in SampEn were observed (*p*>0.01); and 3) lower RQAEn was observed in the signal with 40 units of noise compared to the signals with 120, 160 and 200 units of noise (*p*<0.01), no other changes in RQAEn were observed (*p*>0.01). Follow-up tests for the main effect of sampling frequency revealed the following: 1) ApEn systematically decreased as sampling frequency decreased (*p*<0.01); 2) SampEn was not affected by sampling frequency (*p*>0.01); and 3) higher RQAEn was observed in the 500 Hz signal compared to the 250, 200 and 166 Hz signals and a higher RQAEn for the 333 Hz signal compared to the 200 Hz signal (all *p*>0.01); no other RQAEn differences were observed (*p*<0.01).

### Influence of sampling frequency independent of noise

A significant effect of sampling frequency was found for ApEn (*F*_4,20_ = 18.59, *p*<0.01), SampEn (*F*_4,20_ = 192.22, *p*<0.01) and RQAEn (*F*_4,20_ = 6.65, *p* = 0.03) in the original CoP data with no noise added. Follow-up tests revealed: 1) lower ApEn was observed in the 500 Hz signal compared to all other signals (*p*<0.01), no other differences in ApEn were observed (*p*>0.01); 2) SampEn did not differ between the 250 Hz and 200 Hz signal (*p* = 0.077) or the 200 Hz and 166 Hz signal (*p* = 0.014), but increased as sampling frequency decreased in all other signals (*p*<0.01); and 3) higher RQAEn was observed for the 500 Hz signal compared to the 250 Hz signal (*p*<0.01) with no further observed differences in RQAEn (*p*>0.01).

A significant effect of sampling frequency was also observed in the increment CoP data when calculating ApEn (*F*_4,20_ = 267.72, *p*<0.01). However, no effect was observed when calculating either SampEn (*p* = 0.70) or RQAEn (*p* = 0.64). The follow up test revealed that ApEn systematically decreased as sampling frequency decreased (*p*<0.01). Entropy estimations of the original and increment CoP data at all sampling frequencies are found in Table 3.

**Table 3:** Approximate Entropy (ApEn), Sample Entropy (SampEn) and Recurrence Quantification Analysis Entropy (RQAEn) values for the original and increment center of pressure (CoP) data as a function of sampling frequency.

	Sampling frequency	ApEn	SampEn	RQAEn
*Original CoP*	500 Hz	0.37±0.11	0.40±0.13	4.69±0.76
	333 Hz	0.43±0.11	0.51±0.13	4.60±0.79
	250 Hz	0.46±0.10	0.60±0.15	4.57±0.74
	200 Hz	0.45±0.10	0.63±0.13	4.51±0.73
	166 Hz	0.43±0.10	0.68±0.14	4.46±0.75
*Increment CoP*	500 Hz	1.28±0.02	2.05±0.04	0.96±0.03
	333 Hz	1.09±0.02	2.08±0.04	0.96±0.05
	250 Hz	0.96±0.03	2.06±0.05	0.97±0.05
	200 Hz	0.83±0.04	2.08±0.07	0.98±0.06
	166 Hz	0.76±0.05	2.09±0.11	0.94±0.10

## Discussion

Three main findings were observed. First, increased noise in the synthetic signals and original CoP data caused an increase in SampEn, but a decrease in RQAEn. Second, the degree to which sampling frequency (and time series length) influenced entropy in the original CoP data varied between techniques. Third, the increment CoP data was less influenced by both noise and sampling frequency. Each of these main findings is further discussed below.

### Effects of Added Noise

All three entropy measures were differently influenced by noise. This is a particularly important finding as noise is inherent in all biophysical signals, of which the origin may be mechanical (due to the properties of the recording equipment) or physiological (due to various levels of neural noise). For example, different labs use different force platforms when collecting CoP data [30] while others compare populations with potentially different neural noise levels [31]. Therefore, it is important to understand how different levels of noise, sampling frequency, and time series length influence various estimates of entropy.

We found that adding noise to the synthetic signals resulted in distinct results for each entropy calculation. First, ApEn exhibited an inverted-U pattern as the synthetic signal was shifted from chaotic to stochastic, which leads to the interpretation that the signal moderately contaminated by noise was the most complex. SampEn was more robust to additive noise, as previously reported in a study examining cardiac signals [5]. Specifically, SampEn linearly increased as stochastic noise was added to the signal. Unlike the ApEn results, these findings are intuitive as a SampEn value of 0 is indicative of a regular pattern (e.g., a sine wave) and values close to 2 indicate a more irregular, complex pattern.

Interestingly, RQAEn patterns were very different from both ApEn and SampEn. Specifically, RQAEn decreased as stochastic noise in the signal was increased. This finding is not intuitive since a decrease in entropy is typically believed to occur as a signal becomes more regular or sinusoidal. This finding is consistent with previous research that has shown RQAEn fluctuates and produces non-intuitive results when noise is added to a signal. Specifically, Pellechia and Shockley [32] reported that RQAEn decreased when comparing a complex signal (Lorenz attractor: 4.8 bits/bin) to a regular signal (sine wave: 1.9 bits/bin) and further decreased when noise was added to a sine wave (0.3 bits/bin). The current study extends past research by showing that added noise results in decreased RQAEn in both a physiological and synthetic signals.

**Figure 4:**
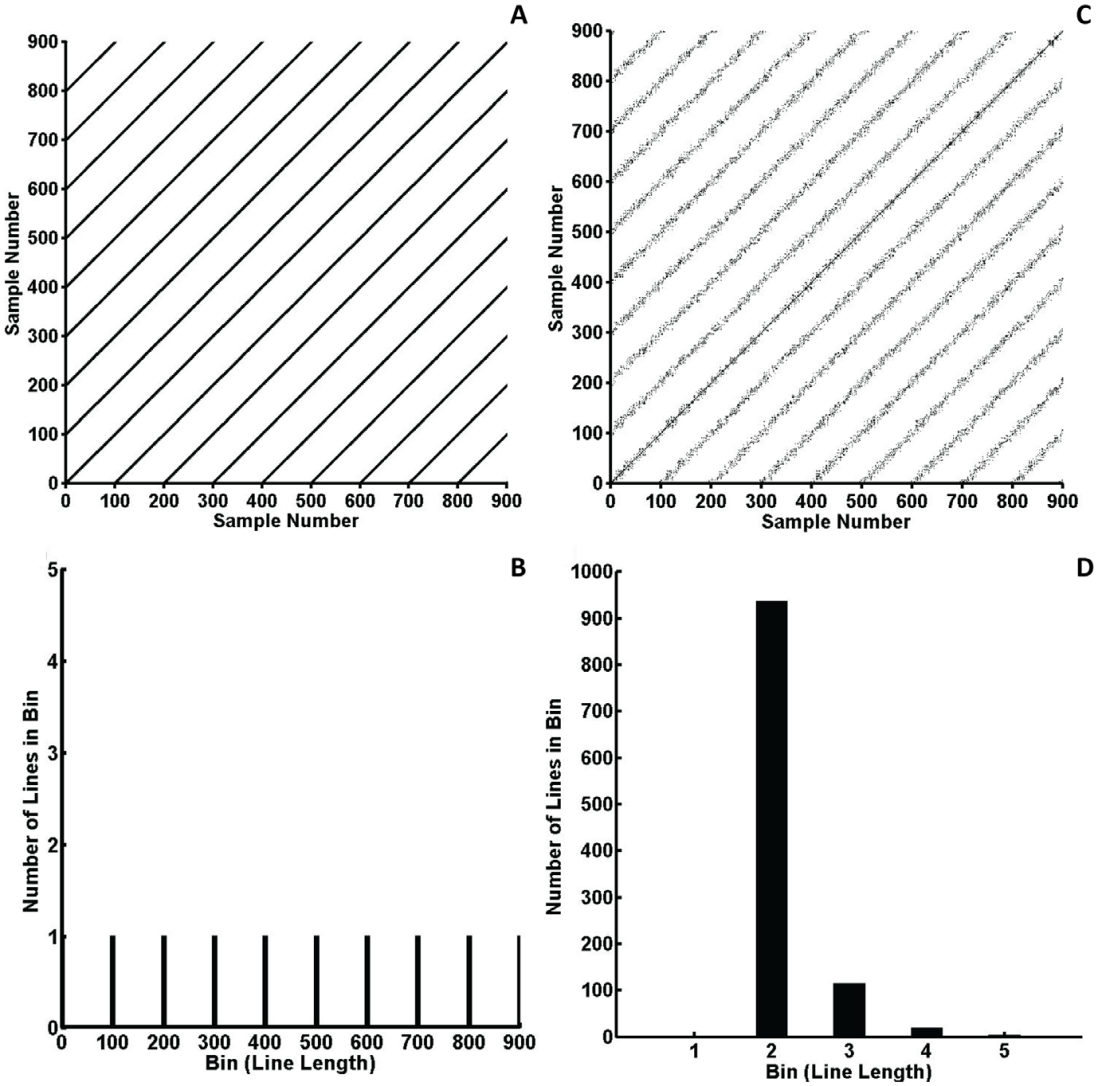
Recurrence plots of sine waves. Recurrence plot (A) and entropy histogram (B) of a sine wave collected at 166 Hz with no noise added. Recurrence plot (C) and entropy histogram (D) of a sine wave collected at 166 Hz with noise added (scaling factor (µ) = 200).

The reason RQAEn produces these counterintuitive results arises from the manner in which it is calculated from the recurrence plot. To illustrate, the recurrence plots of two sine waves are presented in Figure 4; one without noise and one with 200 units of noise. The sine wave with no noise forms long diagonal line segments that contain many points (Figure 4A). The result is a recurrence plot that does not contain many line segments, but the few line segments it does contain vary in length (Figure 4B). Since RQAEn is based on the number of different diagonal line lengths, the sine wave produced a relatively high RQAEn. However, when white noise was added, the long line segments were broken up (Figure 4C). The recurrence plot for the noisy sine wave contained many more line segments that were, for the most part, only two points long (Figure 4D). As a result, RQAEn was reduced simply because of the high consistency in the length of the line segments. These results suggest that caution should be taken when using RQAEn to measure the complexity of a signal, as changes in entropy may be due to noise or signal regularity factors. Also, it should be noted that unlike ApEn and SampEn, RQAEn assesses the complexity of the recurrence plot rather than the complexity of the original signal. Thus, extending RQAEn results back to the original signal can result in erroneous interpretations.

The effects of noise on the CoP data were similar to those of the synthetic signals. In general, ApEn and SampEn tended to increase as noise was added to the signal. For RQAEn, an inverse relationship was again observed, with increased noise leading to lower RQAEn. Yet, these estimates and effects can be confounded by differences in sampling frequency, which varies widely between studies of postural control and other physiological systems.

### Sampling Frequency Effects

Changes in sampling frequency can influence entropy calculations in two ways. Firstly, as sampling frequency increases, movement dynamics over smaller time scales are captured. Secondly, with increases in sampling frequency, more data points are included in the final data sample. The influence of sampling frequency when calculating ApEn, SampEn and RQAEn was examined by downsampling the original 1000 Hz CoP signal, producing five new signals with different effective sampling rates. With the exception of the signal with the highest sampling frequency (500 Hz), both ApEn and RQAEn were robust across all sampling frequencies. SampEn, however, showed significant differences across all sampling frequencies in both the noise free CoP signal and in the CoP signal with added noise. This was surprising since SampEn has been reported to be unaffected by time series length in cardiac signals [5]. Care should therefore be taken when comparing SampEn between studies where the CoP time series length and sampling frequencies are different.

The observed decrease in SampEn at higher frequencies suggests SampEn is more sensitive to the co-linearities that are present in an oversampled signal. Co-linearities occur when a high sampling rate (well above the Nyquist) is used to capture a low frequency movement (e.g., CoP). These co-linearities ultimately lead to a decrease in entropy because the signal appears overly regular due to an increase in the number of matches to the template pattern. In this case, the decrease in entropy is an artifact of the sampling rate and does not reflect the underlying control process. Interestingly, ApEn and RQAEn were not affected by an increase in sampling frequency until the 500 Hz threshold, suggesting that co-linearities do not affect these measures at lower sampling frequencies. As previously mentioned, this finding is counter to other papers in the literature that have suggested SampEn is less sensitive to time series length than ApEn (comparisons between SampEn and RQAEn have not been reported in previous literature). The discrepancies between this study and previous studies likely resulted from the way time series length was manipulated. In the previous studies, data was collected for a longer period of time to increase the length of the data series. This suggests that SampEn may be more robust to shorter time series when co-linearities are not an issue. However, when the length of the time series is determined by altering the sampling rate, SampEn may produce artifacts in the final entropy measure. This is an interesting finding given that different labs routinely collect CoP data at different frequencies. When comparing results between studies researchers should therefore consider the sampling frequency that was used.

Thus, it appears that sampling frequency rather than number of data points is the primary concern when calculating entropy from CoP data. This fact is important since previous research has indicated that trials with a small amount of data points can produce erroneous entropy results. Thus, it would be tempting for researchers to sample data at excessively high frequencies to obtain a sufficiently large number of data points, as opposed to collecting data for a prolonged period of time. While the above is true, there is the caveat in the study of nonlinear biological systems. Extending the sampling period could in itself introduce different dynamics arising from the system entering into a new state, so it is imperative that an appropriate sample duration be utilized so that a large enough window is used to analyze the system's dynamics. The results presented above can, however, be used as a guide to help researchers determine the maximum sampling rate for entropy calculations.

### Effects of Long-Range Correlations

A further potential confound when assessing the entropy of biological signals is the presence of long-range correlations in the time series. The validity of complexity estimates in such signals has been called into question, as the presence of long-range correlations may mask the underlying dynamics of the system [18]. To remove the long-range correlations found in the CoP time series, an increment method has been employed [17], [18]. Using DFA, our data show that the increment method was successful at removing the long-range correlations. This resulted in a decreased effect of sampling frequency when calculating SampEn and RQAEn in the increment CoP data in comparison to the original CoP data. Similarly, the effect of added noise was minimized in all measures of entropy when increment CoP data was used. These results suggest that long-range correlations should be removed from CoP data prior to calculating entropy as they can confound the interpretation of the entropy results.

### Theoretical and practical reflections on entropy comparisons

The data presented in this paper is not intended to suggest that measures of entropy are impractical or too difficult to correctly employ. Rather, it has often been shown that these techniques provide information about the health, stability, flexibility and adaptability of the postural system that is not captured using traditional techniques. It should also be noted that many of the issues raised in this paper relate to making between-study comparisons. However, employing a within-study comparison allows researchers to compare the dynamics of behavior across different subject groups and/or experimental conditions and is less susceptible to the aforementioned issues. Within a study, sampling rate, data collection length and mechanical noise are typically held constant and therefore cancel out across conditions. Thus, relative comparisons can be confidently made.

While making relative comparisons may resolve many of the issues raised in this paper and allow for the identification of how an experimental condition influences the dynamics of behavior, such comparisons are not necessarily beneficial to a clinician or practitioner. For example, it has been shown that the ApEn of a knee angle trajectory time series during treadmill locomotion decreases following an ACL injury [33]. However, the significance of this data is derived from a between-subjects comparison (i.e., healthy controls vs. ACL-injured subjects) rather than from a relative comparison using a within-subjects design (i.e., pre/post injury). Since the range of “healthy” gait dynamics vary from individual to individual, even within an uninjured population, it is impossible to know exactly how an injury affects a specific individual's behavior without having baseline measures (i.e., pre-injury behavior). Similar issues are present when comparing patients with a neurological disorder relative to non-afflicted individuals. In both cases, biological noise inherent to the disorder could be present, resulting in differences between subject populations. When these types of studies are conducted, the results from this study could be used as a guideline to help researchers appropriately calculate entropy. As the field moves forward, especially in the rehabilitation and medical domains, it will become imperative that boundaries in the metrics that describe “healthy” and “unhealthy” behavior are identified. Furthermore, the development of consistent measurement and analysis techniques will be essential to the creation of normative data sets.

Aside from practical issues, theoretical implications should also be taken into consideration. Our findings demonstrate that while comparisons across groups and experimental conditions can be made, much still remains to be understood regarding the properties of biological signals. Understanding the underlying source of dynamic complexity and noise (both internal and external) within biological signals, such as postural sway, remain essential to gaining insight into the underlying connections between physiological systems and the pathophysiology of disease and disorder.

### Summary

A major driving force in the literature has been the view that a decrease in entropy supports the loss-of-complexity hypothesis [4] which states that a frail or diseased system exhibits a less complex pattern. Evidence for this hypothesis has been observed in heart rate [34], blood pressure [4] and stride-to-stride intervals during gait [33]. However, it has also been proposed that increased neural noise underlies behavioral differences [9]–[11] and age-related deficits in performance [12]. The findings in the current study illustrate the difficulty in separating complexity from randomness in a physiological signal that can be contaminated by internal and external sources of noise.

Factors such as noise level and sampling frequency can affect the estimation of entropy and caution should be exhibited when interpreting different entropy estimates. For this reason, an increase in entropy cannot generally be interpreted as a reflection of the same physiological changes across all studies. Estimations of entropy can be useful clinical tools to identify levels of adaptability during an assessment and/or rehabilitation program [35]. It is especially important to understand how the estimate of entropy can change within a specific population, as it can potentially lead to insight into the mechanisms of a disorder or lead to novel clinical interventions.
